# Novel use of a bronchoscope and bariatric needle holder for retrieval of retained intramedullary metalwork during total knee arthroplasty

**DOI:** 10.1016/j.mex.2023.102080

**Published:** 2023-02-15

**Authors:** Emmanuel Olusola Oladeji, Stanley Masunda, Ahmed Lashin, Oghofori Obakponovwe, Madhu Rao

**Affiliations:** Department of Trauma and Orthopaedics, St. Richard's Hospital, Chichester, United Kingdom

**Keywords:** Retained metalwork, Retained pin, Total knee replacement, Intramedullary referencing, Novel use of a bronchoscope and bariatric needle holder for retrieval of retained intramedullary metalwork during total knee arthroplasty

## Abstract

Retained metalwork during total joint arthroplasties usually occur from accidental misplacement of jig locking pins into the medullary canal via the aperture created for intramedullary referencing. They are associated with significant clinical and financial consequences for the patient, surgeon and health care provider. Hence the imperative to device methods to not only prevent their occurrence but reliably retrieve any trapped foreign body. We describe an easy, reliable, reproducible, fluoroscopy-free and time efficient way to retrieve metalworks trapped in the medullary canal, with the aid of a bronchoscope and a bariatric needle holder.

• The described method utilises two instruments which are readily available in theatres – single use flexible bronchoscope and bariatric needle holder.
• Prior experience in bronchoscopy or laparoscopic surgery is not required to adapt the instruments for this purpose.
• This technique minimises both the stress on the patient and the surgical team.

## Graphical abstract

**Figure:**
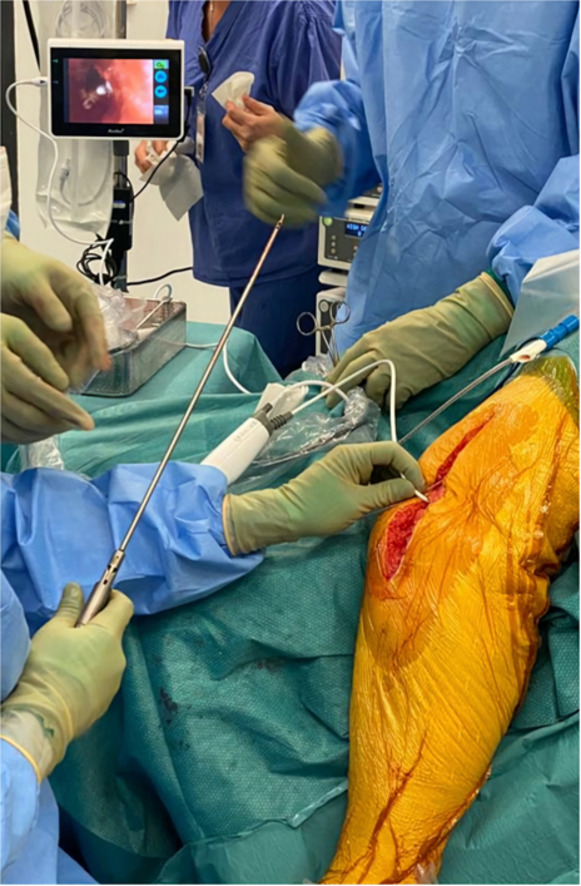

Specifications table

**Table:** 

Subject area:	Medicine and Dentistry
More specific subject area:	Orthopaedics
Name of your method:	Novel use of a bronchoscope and bariatric needle holder for retrieval of retained intramedullary metalwork during total knee arthroplasty
Name and reference of original method:	N.A
Resource availability:	N.A

**Method details**

## Background

Total knee replacements (TKRs) are commonly performed for end-stage knee osteoarthritis. Complications of retained metalwork during TKRs are scarcely reported. Typically, this involves a jig locking pin, accidentally misplaced into the medullary canal of the femur via the aperture created for intramedullary referencing. If retained, foreign bodies pose a risk of leading to further complications, can damage the patient-surgeon relationship and significantly increase post-operative paperwork [1]. Generally, patients who experienced retained foreign bodies after total joint arthroplasty (TJA) surgeries tend to undergo additional procedures, stay longer in the hospital and experience more complications, with associated financial consequences for both the patient and the health care provider [2].

## Technique

**Fig. 1:**
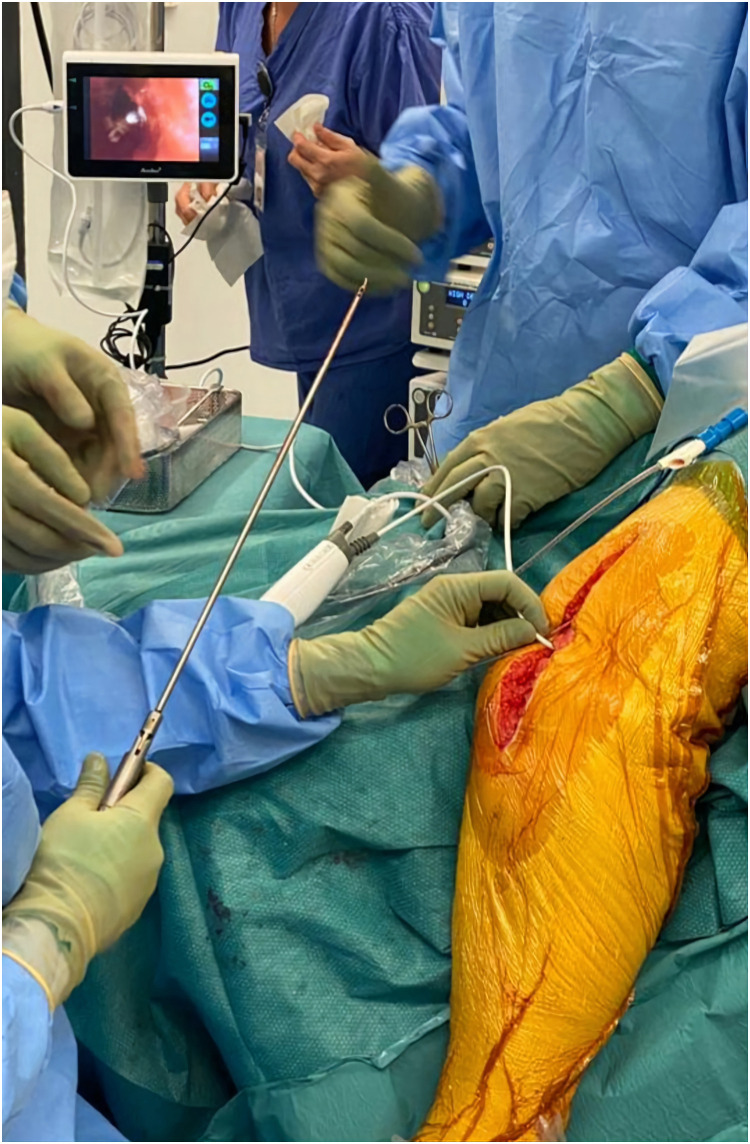
Image demonstrating visualisation of the trapped metalwork on the monitor connected to the bronchoscope, with the bariatric needle holder being held in the surgeon's right hand for retrieval of the pin under direct vision.

Our technique utilises a sterile single use video bronchoscope, the Ambu^R^  aScope^TM^ and a bariatric needle holder. These instruments are readily available in theatres, as they are frequently used by anaesthetists and general surgeons. The Ambu^R^  aScope^TM^ connects to a HD monitor and gives impeccable views of the medullary canal after insertion, with a variable 180-degree arc of tip angulation, including flush and suction capabilities. The bariatric needle holder also of narrow calibre, allows easy insertion into the medullary canal, is easy to manoeuvre and is of adequate length to span the femur. The retrieval is undertaken with patient lying supine, operating table inclined to 30˚ and the involved leg dropped over the side of the table. The bronchoscope is carefully guided into the medullary canal to visualise the misplaced metalwork and retrieved using a bariatric needle. Using both instruments, the retained pin gets retrieved under direct vision in minutes (Fig. 1). These two instruments can be conveniently repurposed for this intervention without prior experience in bronchoscopy or laparoscopic surgery. This technique has been adopted in our practice – in appropriate clinical contexts, as found to be reliable and time efficient.

## Discussion

Publications on this subject are rare. Yau et al reported an incidence of 1% over a 6-year period while Van Doren and colleagues found retained foreign bodies in 0.01% of all TJA procedures from a national study [2,3]. Various strategies to retrieve retained pins have been described; the gravity method of hanging the leg over the table, the use of a suction device, the use of a bent k-wire under fluoroscopic guidance and a magnetized intramedullary rod [2,3]. However, the first 3 strategies from our experience are technically challenging and can push the metalwork further proximal, compounding the problem, while the magnet technique will only be successful if the misplaced metalwork can be effected by a magnetic field [1].

## Conclusion

Our technique is easy, reliable, reproducible, fluoroscopy-free and a time efficient way to retrieve retained intramedullary metalwork, minimising both the stress on the patient and surgical team.

## Ethics statements

The relevant informed consent was obtained.

## CRediT authorship contribution statement

**Emmanuel Olusola Oladeji:** Conceptualization, Writing – original draft, Writing – review & editing. **Stanley Masunda:** Writing – original draft, Writing – review & editing. **Ahmed Lashin:** Conceptualization, Writing – original draft, Writing – review & editing. **Oghofori Obakponovwe:** Conceptualization, Supervision, Writing – review & editing. **Madhu Rao:** Conceptualization, Supervision, Writing – review & editing.

## Declaration of Competing Interest

The authors declare that they have no known competing financial interests or personal relationships that could have appeared to influence the work reported in this paper.

## Data Availability

Data will be made available on request.
